# Testis‐enriched kinesin KIF9 is important for progressive motility in mouse spermatozoa

**DOI:** 10.1096/fj.201902755R

**Published:** 2020-02-19

**Authors:** Haruhiko Miyata, Keisuke Shimada, Akane Morohoshi, Seiya Oura, Takafumi Matsumura, Zoulan Xu, Yuki Oyama, Masahito Ikawa

**Affiliations:** ^1^ Research Institute for Microbial Diseases Osaka University Suita Japan; ^2^ Graduate School of Medicine Osaka University Suita Japan; ^3^ Graduate School of Pharmaceutical Sciences Osaka University Suita Japan; ^4^ Graduate School of Life and Medical Sciences Doshisha University Kyotanabe Japan; ^5^ The Institute of Medical Science The University of Tokyo Tokyo Japan; ^6^ Present address: Institute of Molecular Medicine and Life Science Yokohama City University Association of Medical Science Yokohama Japan

**Keywords:** fertilization, male fertility, sperm motility

## Abstract

Kinesin is a molecular motor that moves along microtubules. Kinesin family member 9 (KIF9) is evolutionarily conserved and expressed strongly in mouse testis. In the unicellular flagellate *Chlamydomonas*, KLP1 (ortholog of KIF9) is localized to the central pair microtubules of the axoneme and regulates flagellar motility. In contrast, the function of KIF9 remains unclear in mammals. Here, we mutated KIF9 in mice using the CRISPR/Cas9 system. *Kif9* mutated mice exhibit impaired sperm motility and subfertility. Further analysis reveals that the flagella lacking KIF9 showed an asymmetric waveform pattern, which leads to a circular motion of spermatozoa. In spermatozoa that lack the central pair protein HYDIN, KIF9 was not detected by immunofluorescence and immunoblot analysis. These results suggest that KIF9 is associated with the central pair microtubules and regulates flagellar motility in mice.

**Abbreviations**
ASH: ASPM‐SPD2‐Hydin
ES: embryonic stem
IVF: in vitro fertilization
Kif9: kinesin family member 9
KO: knockout
LD: large deletion
MEF: mouse embryonic fibroblast
TEM: transmission electron microscopy
VAP: average path velocity
VCL: curvilinear velocity
VSL: straight line velocity
WT: wild type
ZP: zona pellucida

## 1 INTRODUCTION

Spermatozoa are highly specialized cells that are composed of two parts, head, and flagellum. The head contains the nucleus where the paternal genetic information is stored and an acrosome, an exocytotic vesicle that surrounds the nucleus. The flagellum is a motile, thread‐like appendage that can be divided into three parts, midpiece, principal piece, and end piece.^1^, ^2^ The midpiece contains a mitochondrial sheath that plays roles in energy production, whereas the principal piece contains a fibrous sheath that provides elastic rigidity and a scaffold for glycolytic and signaling molecules. These accessory structures are not localized in the end piece.^1^, ^2^ Any defects in the formation or function of these structures could lead to male sterility.

The central component of the flagellum is the axoneme, a “9+2” structure that consists of a central pair of two singlet microtubules surrounded by nine outer microtubule doublets.^3^ In addition to microtubules, there are several macromolecular complexes that compose the axoneme such as outer and inner dynein arms that slide doublet microtubules and radial spokes that are localized between the central pair and doublet microtubules. Molecular components of these structures have been extensively studied in the unicellular flagellate *Chlamydomonas*.^3^ Many proteins identified in *Chlamydomonas* are conserved in mammals including mice and humans; however, their functions and association with infertility in mammals remain to be understood.

Kinesin is a motor protein that moves along microtubules, usually in an anterograde manner. Forty‐five kinesins with varying functions have been found in humans,^4^ which compose the kinesin superfamily of proteins (KIFs). *Kif9* is evolutionarily conserved and its function has been studied in unicellular organisms. In *Chlamydomonas*, KLP1 (ortholog of KIF9) is localized in the central pair of the axoneme.^5^, ^6^ Knocking down *KLP1* leads to a reduction in swimming velocity, suggesting that KLP1 is involved in flagellar motility.^6^ Supporting this idea, knockdown of *KIF9A* (ortholog of *Kif9*) in *Trypanosoma brucei* leads to impaired motility without visible structural abnormalities of their flagella.^7^

In addition to these studies with unicellular organisms, Northern blot analysis using mouse tissues showed that *Kif9* is expressed strongly in the testis,^8^ suggesting that KIF9 is involved in regulating sperm motility. In this study, we confirmed that KIF9 is localized to the mouse sperm flagella. Further, we mutated *Kif9* in mice using the CRISPR/Cas9 system and analyzed its function in male fertility and sperm motility.

## 2 MATERIALS AND METHODS

### 2.1 Animals

All animal experiments were approved by the Animal Care and Use Committee of the Research Institute for Microbial Diseases, Osaka University. Mice were purchased from CLEA Japan (Tokyo, Japan) or Japan SLC (Shizuoka, Japan).

### 2.2 RT‐PCR

Mouse cDNA was prepared from various tissues of adult ICR mice or testes from 1‐ to 5‐week‐old males with SuperScript III First‐Strand Synthesis System (Thermo Fisher Scientific, MA, USA) using an oligo (dT) primer. RT‐PCR was performed using 10 ng of cDNA with the following forward and reverse primers: 5′‐AGAAGGACACTCGGAGAGGG‐3′ and 5′‐CGCGGTGCTTGTAATTCTCC‐3′ for *Kif9*, 5′‐AAGTGTGACGTTGACATCCG‐3′, and 5′‐GATCCACATCTGCTGGAAGG‐3′ for *Actb*. The amplification conditions were 1 minute at 94°C, followed by 35 cycles of 94°C for 30 seconds, 65°C for 30 seconds, and 72°C for 30 seconds, with a final 1‐minute extension at 72°C.

### 2.3 In silico data analysis

Single cell transcriptome data in the mouse testis that was published previously^9^ was obtained. *Kif9* expression in those cells was analyzed using Loupe Cell Browser 3.3.1 (10× Genomics, CA, USA).

### 2.4 Immunofluorescence

Spermatozoa collected from the cauda epididymis were diluted in PBS, spotted onto slides, air‐dried, fixed with 4% paraformaldehyde for 10 minutes, and washed in PBS for 5 minutes. The slides were blocked with 5% BSA and 10% goat serum in PBS for 1 hour at room temperature. The slides were then incubated with rabbit anti‐KIF9 antibody (1:50, #HPA022033, Atlas Antibodies, Bromma, Sweden) overnight at 4°C and washed with PBS three times for 10 minutes each. After incubation with Alexa Fluor 488 or Alexa Fluor 546‐conjugated secondary antibody (1:200, #A11070 or #A11071, Thermo Fisher Scientific) at room temperature for 2 hours, the slides were washed with PBS three times for 10 minutes each. The slides were then incubated with Hoechst 33342 (2 µg/mL) (Thermo Fisher Scientific) for 15 minutes and washed with PBS three times for 10 minutes each. Slides were observed with an Olympus BX‐53 microscope (Tokyo, Japan).

### 2.5 Sperm protein fractionation

Sperm protein fractionation was performed as described previously.^10^, ^11^ Spermatozoa obtained from the cauda epididymis were suspended in 1% Triton X‐100 lysis buffer (50 mM NaCl, 20 mM Tris‐HCl, pH 7.5, protease inhibitor mixture) and incubated for 2 hours at 4°C. The sample was centrifuged at 15 000 *g* for 10 minutes to separate the Triton‐soluble fraction and the Triton‐resistant fraction. The pellet (Triton‐resistant fraction) was resuspended in 1% SDS lysis buffer (75 mM NaCl, 24 mM EDTA, pH 6.0) and incubated for 1 hour at room temperature. The sample was centrifuged at 15 000 *g* for 10 minutes to separate the SDS‐soluble fraction and SDS‐resistant fraction. The pellet (SDS‐resistant fraction) was resuspended in sample buffer (66 mM Tris‐HCl, 2% SDS, 10% glycerol and 0.005% Bromophenol Blue), boiled for 5 minutes, and centrifuged at 15 000 *g* for 10 minutes.

### 2.6 Immunoblot analysis

Immunoblot analysis was performed as described previously.^12^ Samples were subjected to SDS‐PAGE followed by western blotting. After blocking with 10% skim milk, blots were incubated with primary antibodies overnight at 4°C and then incubated with secondary antibodies conjugated to horseradish peroxidase (1:10,000, #805‐035‐180, #111‐036‐045, #115‐036‐062, or #112‐035‐167, Jackson ImmunoResearch, PA, USA) for 2 hours at room temperature. Antibodies used: goat anti‐KIF9 1:100 (#SC99958, Santa Cruz Biotechnology, CA, USA); rabbit anti‐ACTB 1:1000 (#PM053, Medical & Biological Laboratories, Aichi, Japan); goat anti‐BASIGIN 1:500 (#SC9757, Santa Cruz Biotechnology), mouse anti‐acetylated tubulin 1:1000 (#T7451, Sigma‐Aldrich, MO, USA); mouse anti‐AKAP4 1:5000 (#611564, BD Biosciences, CA, USA); mouse anti‐phosphotyrosine 1:1000 (#05‐321, Merck Millipore, MA, USA); rabbit anti‐RSPH9 1:200 (#HPA031703, Atlas Antibodies); rat anti‐PA 1:1000 (#012‐25863, FUJIFILM Wako Pure Chemical, Osaka, Japan); and rabbit anti‐FLAG 1:1000 (#PM020, Medical & Biological Laboratories). Immunoreactive proteins were detected by an ECL western blotting detection kit (GE Healthcare, Little Chalfont, UK).

### 2.7 gRNA design

gRNAs with fewer off‐target sites were found using the online source CRISPRdirect.^13^ The gRNA sequence for an indel mutation was 5′‐TCATGAGCAAAGTCATCAGT‐3′ (exon 2) and target sequences for a large deletion were 5′‐TAAAATGGGTACTAGGAAAA‐3′ (exon 2) and 5′‐AGCAGCTCTAGTCTGTTCTA‐3′ (exon 21).

### 2.8 Generation of *Kif9* mutant mice (indel)

Superovulated B6D2F1 females were mated with B6D2F1 males and fertilized eggs were collected. Circular pX330 plasmids^14^, ^15^ were injected into one of the pronuclei at 5 ng/µL. The injected zygotes were cultured in KSOM medium^16^ for one day. Two‐cell embryos were then transferred into the oviduct of pseudo‐pregnant ICR mice. Obtained pups were genotyped by PCR and Sanger sequencing.

### 2.9 Generation of *Kif9* mutant mice (large deletion)

*Kif9* large deletion mice were generated using ES cells as described previously.^17^ Briefly, the EGR‐G01 ES cells (1 × 10^3‐4^)^18^ were cultured on mouse embryonic fibroblasts (MEF) in a 6‐well plate and transfected with pX330 targeting exon 2 (1.0 µg) and PX459 targeting exon 21 (1.0 µg) using Lipofectamine LTX & PLUS (Thermo Fisher Scientific). After 14‐18 hours, the cells were selected with puromycin (0.1 µg/mL) for 48 hours, passaged, cultured for 5‐6 more days, picked, and transferred onto MEF cells in 96‐well plates. After 48‐72 hours of culture, each ES cell clone was genotyped. The mutant ES cell clones with normal karyotypes were injected into 8‐cell ICR embryos and the blastocysts were transplanted into the uteri of pseudo‐pregnant ICR females. Obtained chimeric mice were mated with B6D2F1 females to obtain the next generation through germline transmission.

### 2.10 Genotyping

Genotyping was performed with PCR. For the indel mutation, “primer a” (5′‐CACAAAGCAGCTGAAAGACAGG‐3′) and “primer b” (5′‐CTCCACCATTCGGATGGAGG‐3′) were used for PCR and the PCR product was digested with StuI. For large the deletion, “primer a” and “primer b” were used for the WT allele and “primer a” and “primer c” (5′‐TTCTGTGAAGAGGAGCAAGG‐3′) were used for the large deletion allele.

### 2.11 Mating tests

Sexually matured male mice were individually caged with two 8‐week‐old B6D2F1 female mice for 2 months and plugs were checked every morning. The number of pups was counted on the day of birth.

### 2.12 Histological analysis of testis

PAS staining of testis sections was performed as previously described.^19^ The sections were observed with an Olympus BX‐53 microscope.

### 2.13 In vitro fertilization (IVF)

IVF was performed as described previously.^20^ Briefly, spermatozoa collected from cauda epididymis were incubated in TYH medium^21^ for 2 hours at 37°C under 5% CO_2_. Eggs collected from superovulated females were treated with 330 µg/mL of hyaluronidase (Sigma‐Aldrich) for 10 minutes to remove the cumulus cells (cumulus‐free eggs) or with 1 mg/mL of collagenase (Sigma‐Aldrich) for 10 minutes to remove the zona pellucida (ZP) (zona‐free eggs). The incubated spermatozoa were added to a drop of the TYH medium containing intact, cumulus‐free, or zona‐free eggs at a final density of 2 × 10^5^ spermatozoa/mL. When IVF was performed using intact or cumulus‐free eggs, two‐cell embryos were counted the next day. When IVF was performed using zona‐free eggs, the pronuclear formation was observed 6 hours after insemination. For the ZP binding assay, cumulus‐free eggs were incubated with spermatozoa at a density of 2 × 10^5^ spermatozoa/mL and eggs were observed under an Olympus IX‐73 microscope.

### 2.14 Isolation of sperm proteins for tyrosine phosphorylation

Spermatozoa collected from the cauda epididymis were incubated in TYH medium for 10 minutes or 2 hours. Spermatozoa were then collected in PBS and centrifugated at 2000 *g* for 2 minutes at room temperature. The collected spermatozoa were resuspended in sample buffer, boiled for 5 minutes, and centrifuged at 15 000 *g* for 10 minutes. Immunoblot analysis was performed as described above using 5% BSA instead of 10% skim milk for blocking.

### 2.15 Sperm motility analysis

Sperm motility was analyzed as described previously.^22^ Spermatozoa obtained from cauda epididymis were incubated in the TYH medium. Sperm motility was analyzed using the CEROS sperm analysis system (Version 12.3; Hamilton Thorne Biosciences, MA, USA). Analysis settings were as described previously.^23^ For tracing sperm waveforms, spermatozoa were observed with an Olympus BX‐53 microscope equipped with a high‐speed camera (HAS‐L1, Ditect, Tokyo, Japan). The motility was videotaped at 200 frames per second or 50 frames per second. Obtained images were analyzed for waveforms using the sperm motion analyzing software (BohBohsoft, Tokyo, Japan).^24^

### 2.16 Transmission electron microscopy (TEM)

Cauda epididymis samples were prepared for TEM analysis as described previously.^25^ Sections were examined using a JEM‐1400 plus electron microscope (JEOL, Tokyo, Japan) at 80 kV with a CCD Veleta 2K × 2X camera (Olympus).

### 2.17 Generation of *Hydin* KO chimeric mice

*Hydin* KO ES cells that were established previously^26^ were injected into 8‐cell ICR embryos. Obtained blastocysts were transplanted into the uteri of pseudo‐pregnant ICR females.

### 2.18 Generation of KIF9 and HYDIN recombinant proteins

*Kif9* was amplified from mouse testis cDNA, digested with BamHI and EcoRV, and ligated into the FLAG‐tagged (C‐terminus) pCAG vector that contains the CAG promoter and a rabbit globin poly (A) signal.^27^ Primers that were used to amplify the cDNA were 5′‐AAGGATCCGCCGCCATGGGTACTAGGAAAAAGGTTCAAGC‐3′ and 5′‐AAGATATCTTTTCTGTGTGACTGTTGGAGG‐3′. *Hydin* was also amplified from mouse testis cDNA, digested with EcoRV and NheI, and ligated into the PA‐tagged (C‐terminus) pCAG vectors. Primers used were 5′‐AAGATATCGCCGCCATGACCCTGAAGATCAAATGTGTGG‐3′ and 5′‐AAGCTAGCGCTGGTTTCCTGCTTTTCCTCC‐3′ for *Hydin* #1 (1‐408), 5′‐AAGATATCGCCGCCATGATCCTTGAAGACAGCG‐3′ and 5′‐AAGCTAGCCCCACAGGGGGAGGGGCTGGAGAGCAGC‐3′ for *Hydin* #2 (409‐800), and 5′‐AAGATATCGCCGCCATGGTCATCTCCCCCCACAGCACTGTGAGC‐3′ and 5′‐AAGCTAGCCACCTCAAAGCTGAGGTTGG‐3′ for *Hydin* #3 (801‐1218).

### 2.19 Co‐immunoprecipitation

Plasmids were transiently transfected into HEK293T cells and cultured for 24 hours. Immunoprecipitation using harvested cells was performed as previously described.^19^ FLAG M2 antibody (#F1804, Sigma‐Aldrich) was used for immunoprecipitation.

### 2.20 Statistical analysis

Statistical analyses were performed using Student’s *t* test. Differences were considered significant at *P* < .05 (*) or highly significant at *P* < .01 (**) and *P* < .001 (***). Error bars are standard deviation.

## 3 RESULTS

### 3.1 KIF9 is a testis‐enriched protein localized to sperm flagellum

**Figure 1:**
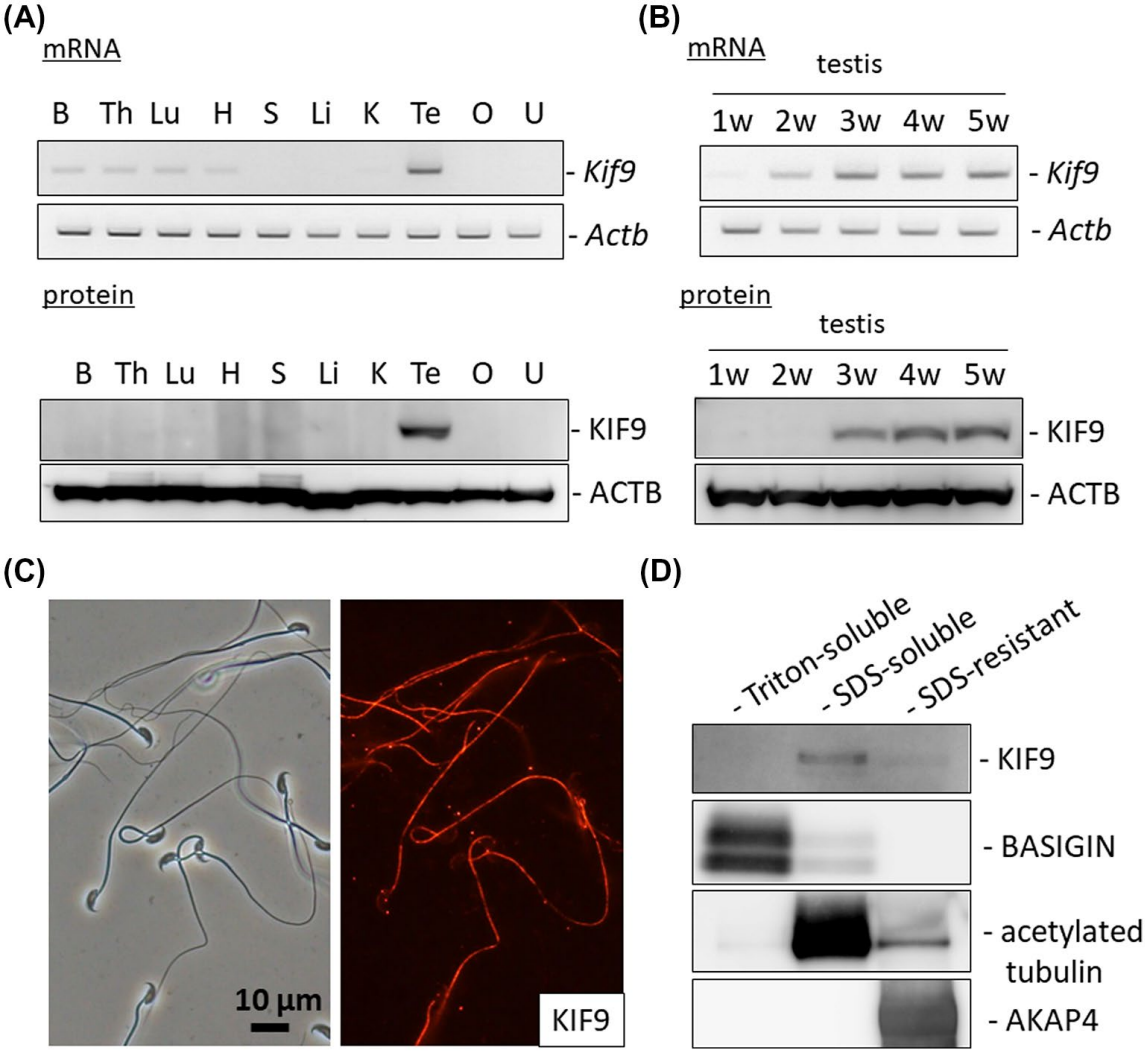
KIF9 is testis‐enriched and localized to mouse flagella. A, Upper, RT‐PCR of *Kif9* using RNAs obtained from various tissues of ICR mice. *Actb* as control. Lower, immunoblot analysis of KIF9 using proteins obtained from various tissues of ICR mice. ACTB as control. B: brain, Th: thymus, Lu: lung, H: heart, S: spleen, Li: liver, K: kidney, Te: testis, O: ovary, and U: uterus. B, Upper, RT‐PCR of *Kif9* using RNAs obtained from various postnatal testes of ICR mice. *Actb* as control. Lower, immunoblot analysis of KIF9 using proteins obtained from various postnatal testes of ICR mice. ACTB as control. C, Localization of KIF9 in spermatozoa. KIF9 is detected in the flagellum. D, Fractionation of mouse spermatozoa. KIF9 was found in the SDS‐soluble fraction. BASIGIN, acetylated tubulin, and AKAP4 were used as makers for the Triton‐soluble, SDS‐soluble, and SDS‐resistant fractions, respectively

RT‐PCR analysis using mouse tissues confirmed that *Kif9* is expressed strongly in the testis with weak expression found in the brain, thymus, lung, and heart (Figure 1A). Western blotting analysis confirmed that KIF9 is expressed strongly in the testis (Figure 1A). Further, RT‐PCR analysis using mouse postnatal testes revealed that *Kif9* starts to express from two weeks, which corresponds to the production of primary spermatocytes. KIF9 protein was detected from three weeks when round spermatids begin to appear (Figure 1B). We confirmed the expression of *Kif9* in spermatocytes and spermatids using an in silico approach by examining an expression database (Supplemental Figure S1). These results suggest that KIF9 may play roles in spermatogenesis and/or fertilization.

Immunofluorescence analysis indicated that KIF9 was localized to the flagellum (Figure 1C). To further analyze KIF9 localization in the flagellum, we fractionated sperm proteins into a Triton X‐100 soluble fraction that contains transmembrane and cytosolic proteins, an SDS‐soluble fraction that contains axonemal proteins, and an SDS‐resistant fraction that contains proteins localized in the accessory structures such as outer dense fibers and fibrous sheath.^10^, ^11^ KIF9 was found in the SDS‐soluble fraction (Figure 1D), suggesting that KIF9 is localized in the axoneme, which is consistent with the studies done in *Chlamydomonas*.^5^, ^6^

### 3.2 *Kif9*‐mutated male mice are subfertile and exhibit partially impaired zona pellucida (ZP) penetration

**Figure 2:**
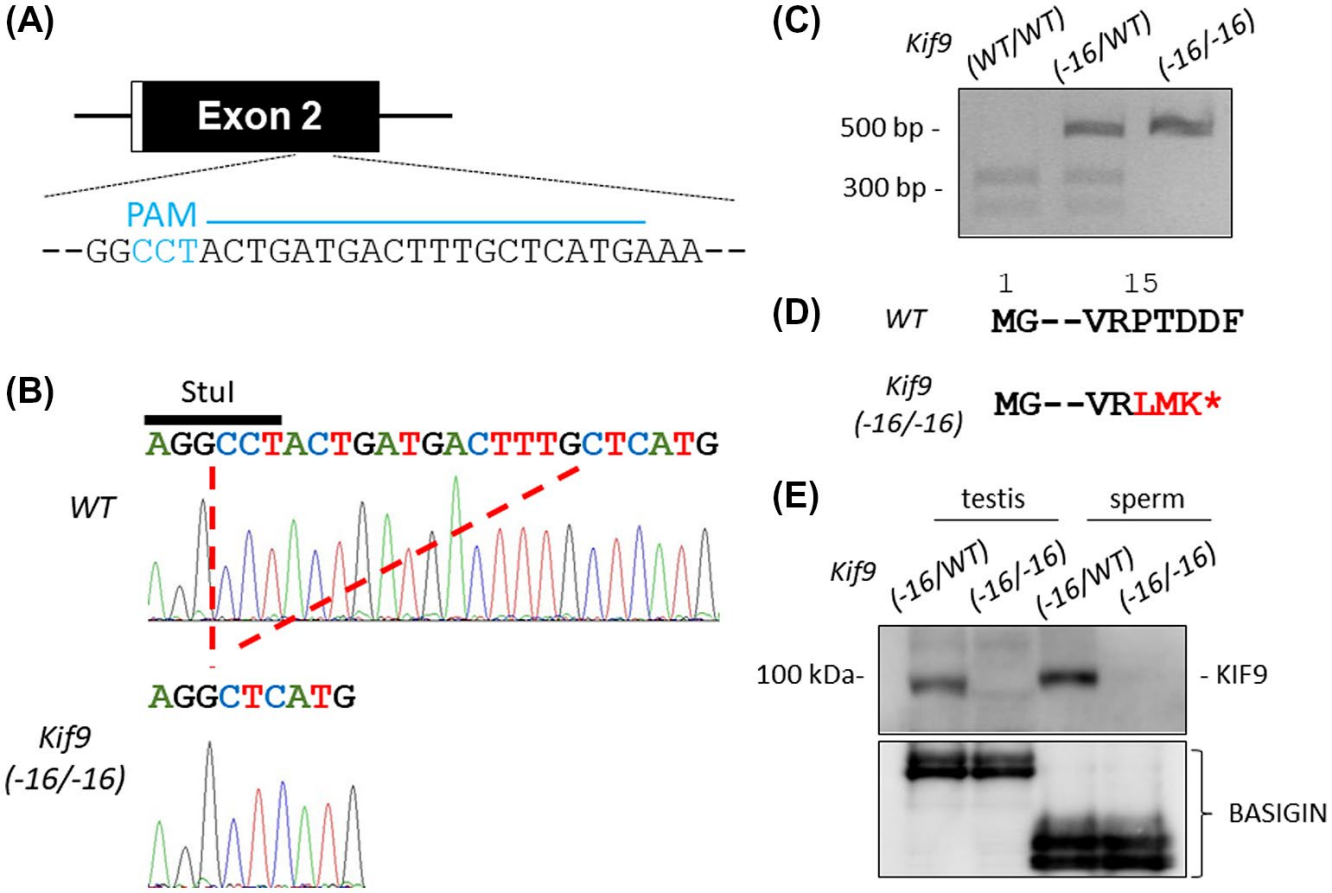
Generation of *Kif9*‐mutant mice. A, CRISPR/Cas9 targeting scheme. gRNA was designed within exon 2 that contains the start codon. Cyan characters indicate PAM (protospacer adjacent motif) sequence. B, Wave pattern sequence of *Kif9*. In mutants, 16 bp nucleotides were deleted. C, Genotyping *Kif9^−16/−16^* mice by StuI digestion. D, The 16 bp deletion caused a P15L mutation resulting in a premature stop codon introduced three amino acids later. E, Protein expression of KIF9 in testis and cauda epididymal spermatozoa. BASIGIN as a loading control

To analyze the function of KIF9 in the spermatozoa, we generated *Kif9*‐mutant mice using the CRISPR/Cas9 system. We injected a pX330 plasmid expressing Cas9 and a gRNA that targets exon 2 (Figure 2A)^14^ into the pronuclei of fertilized oocytes and obtained *Kif9*‐mutant mice that possessed a 16 bp deletion (Figure 2B). Because this deletion disrupts the StuI restriction enzyme site, genotyping can be done by digesting the PCR product with the StuI enzyme (Figure 2C). The 16 bp deletion resulted in a frameshift mutation (P15L) with a premature stop codon introduced three amino acids later (Figure 2D). Obtained *Kif9^−16/−16^* mice did not exhibit overt abnormalities including hydrocephalus that is often observed when the motility of ependymal cilia is impaired.^28^, ^29^ We confirmed that KIF9 was depleted in *Kif9^−16/−16^* male testis and spermatozoa with Western blotting (Figure 2E) and in the null spermatozoa with immunofluorescence (Supplemental Figure S2A). We then analyzed the testis sections of *Kif9^−16/−16^* mice (Supplemental Figure S2B). Although there is a study showing that KIF9 regulates matrix degradation by macrophage podosomes,^30^ no abnormal structures were observed in *Kif9^−16/−16^* testis including spermatogenesis.

**Figure 3:**
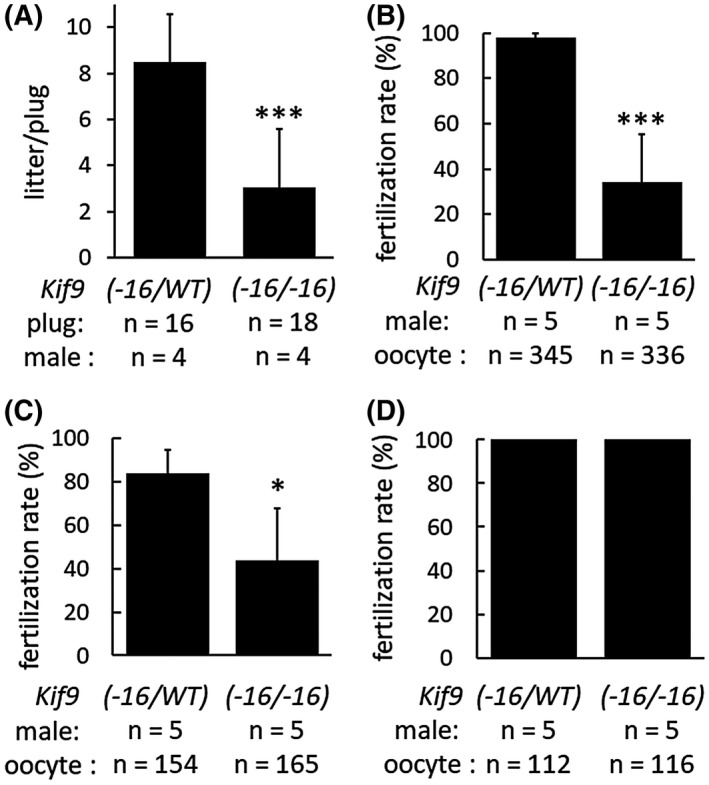
In vivo and in vitro fertility of *Kif9^−16/−16^* male mice. A, Number of litters born per plug detected. n = 4 males each for *Kif9^‐16/WT^* and *Kif9^−16/−16^* mice. B, IVF with cumulus‐intact oocytes. n = 5 males each for *Kif9^‐16/WT^* and *Kif9^−16/−16^* mice. C, IVF with cumulus‐free oocytes. n = 5 males each for *Kif9^‐16/WT^* and *Kif9^−16/−16^* mice. D, IVF with zona pellucida‐free oocytes. n = 5 males each for *Kif9^‐16/WT^* and *Kif9^−16/−16^* mice

Next, to examine fertility, *Kif9^−16/−16^* male mice were mated with wild‐type females for two months and found that homozygous male mice were subfertile (Figure 3A). Further, fewer numbers of eggs were fertilized when we performed in vitro fertilization (IVF) using the spermatozoa from *Kif9^−16/−16^* mice (Figure 3B). Lower fertilization rates in IVF could not be rescued by removing cumulus cells (Figure 3C); however, eggs were fertilized when the ZP was removed (Figure 3D), indicating that ZP penetration is partially impaired in *Kif9^−16/−16^* mice. Although several KO mouse lines exhibit impaired ZP binding,^31^ spermatozoa from *Kif9^−16/−16^* mice could bind to the ZP (Supplemental Figure S3A). We also analyzed the phosphorylation status of tyrosine residues, a hallmark of the capacitation process^32^; however, no differences were observed between *Kif9^‐16/WT^* and *Kif9^−16/−16^* mice (Supplemental Figure S3B).

### 3.3 *Kif9^−16/−16^* **mice exhibit impaired sperm motility**

**Figure 4:**
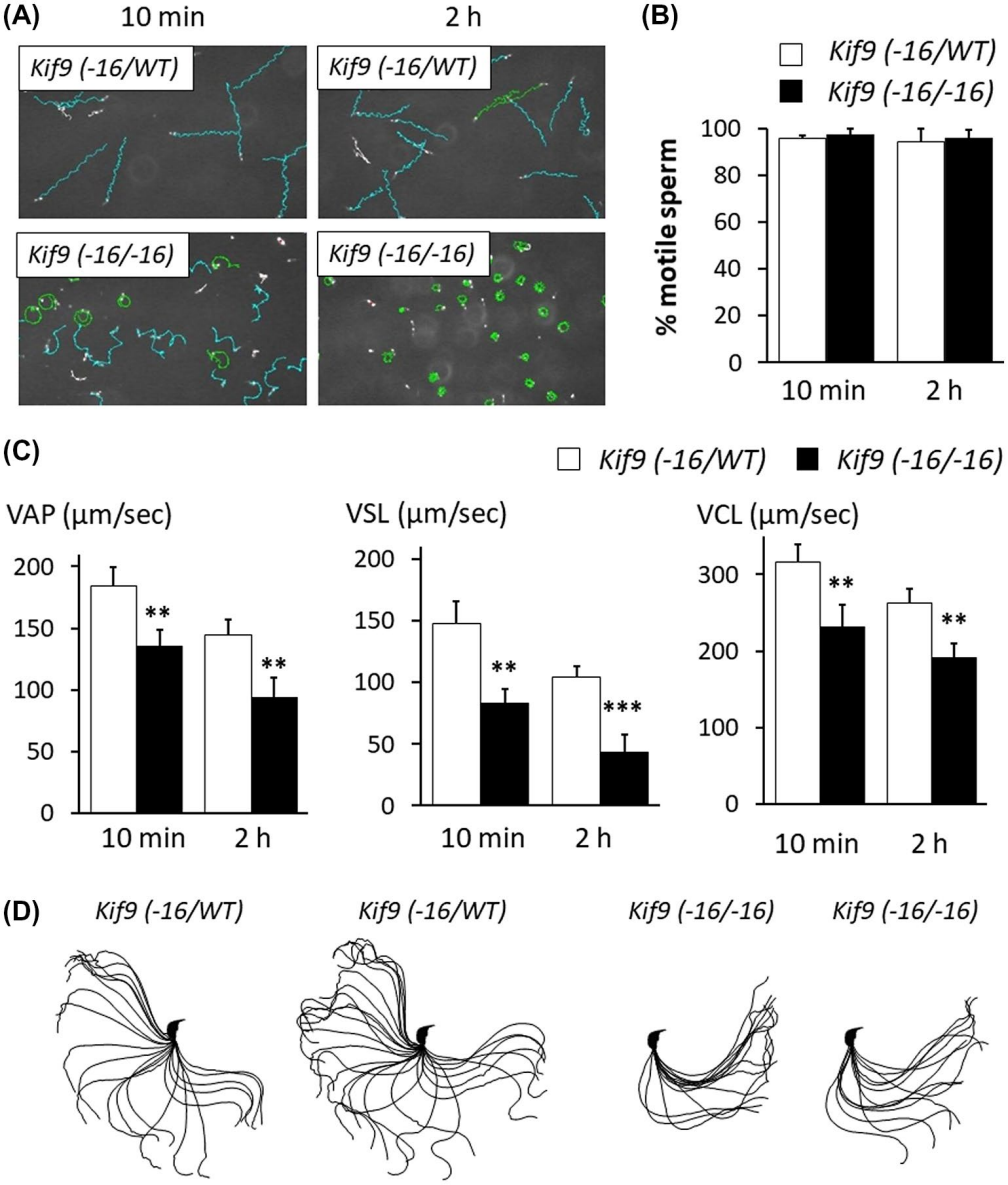
Sperm motility is impaired in *Kif9^−16/−16^* mice. A, Sperm motility tracing performed by a computer‐assisted sperm analysis system after 10 minutes and 2 hours incubation. Cyan tracks were defined as spermatozoa with progressive motility (VSL/VAP ≧ 0.5 and VAP ≧ 50 µm/sec). B, Percentage of motile sperm. n = 4 males each for *Kif9^‐16/WT^* and *Kif9^−16/−16^* mice. C, VAP (average path velocity), VSL (straight line velocity), and VCL (curvilinear velocity) were analyzed. n = 4 males each for *Kif9^−16/WT^* and *Kif9^−16/−16^* mice. D, Flagellar waveforms were analyzed 2 hours after incubation. The motility was videotaped at 200 frames per second. Single frames throughout one beating cycle were superimposed

Localization of KIF9 to the flagellum and partially impaired ZP penetration observed in *Kif9^−16/−16^* mice suggest that KIF9 may play roles in regulating flagellar motility. Therefore, we analyzed sperm motility using a computer‐assisted sperm analysis system. In contrast to the control spermatozoa that move linearly, the trajectory of the moving spermatozoa was circular in *Kif9^−16/−16^* mice (Figure 4A and Supplemental Movies S1, S2), although percentages of motile spermatozoa were comparable between *Kif9^‐16/WT^* and *Kif9^−16/−16^* mice (Figure 4B). Consistent with this observation, velocity parameters such as average path velocity (VAP), straight line velocity (VSL), and curvilinear velocity (VCL) were lower in the *Kif9^−16/−16^* mice than those of *Kif9^‐16/WT^* mice (Figure 4C), indicating that sperm motility is impaired in *Kif9^−16/−16^* mice. To further analyze sperm motility defects, we traced the flagellar waveform (Figure 4D). Flagella of the control spermatozoa could bend to both sides (pro‐hook and anti‐hook)^33^; however, the majority of spermatozoa from *Kif9^−16/−16^* mice could bend only to the side of the hook (pro‐hook) (the number of pro‐hook stall = 105, the number of anti‐hook stall = 30, the number of spermatozoa without stall = 18 out of 153 spermatozoa examined, number of males = 3), which may cause the circular motion of spermatozoa. These results indicate that KIF9 is important in regulating the flagellar waveform pattern.

### 3.4 Generation and phenotypic analysis of *Kif9* “large deletion” mice

Because antibodies used to analyze KIF9 depletion (Figure 2E and Supplemental Figure S2A) recognize the N‐terminus region, there is a possibility that a truncated protein is still produced from a different methionine in *Kif9^−16/−16^* mice. To eliminate the possibility a truncated protein of KIF9 is causing the phenotype observed in *Kif9^−16/−16^* mice, we designed two gRNAs to excise the entire *Kif9* gene, one near the start codon that is different from gRNA used for the 16 bp deletion and another one near the stop codon (Supplemental Figure S4A). In the large deletion (LD) mutant mice, 41 902 bp was deleted and the LD was verified by PCR (Supplemental Figure S4B). *Kif9^LD/LD^* mice did not exhibit overt abnormalities including hydrocephalus, which is consistent with *Kif9^−16/−16^* mice. The depletion of KIF9 in the testis and spermatozoa of *Kif9^LD/LD^* mice was confirmed with Western blotting (Supplemental Figure S4C). *Kif9^LD/LD^* male mice were subfertile (Supplemental Figure S4D) and exhibit impaired sperm motility (Supplemental Figure S4E), as observed in *Kif9^−16/−16^* mice. These results indicate that male subfertility and impaired sperm motility are attributed to the deletion of KIF9.

### 3.5 KIF9 is associated with the axoneme central pair protein HYDIN

**Figure 5:**
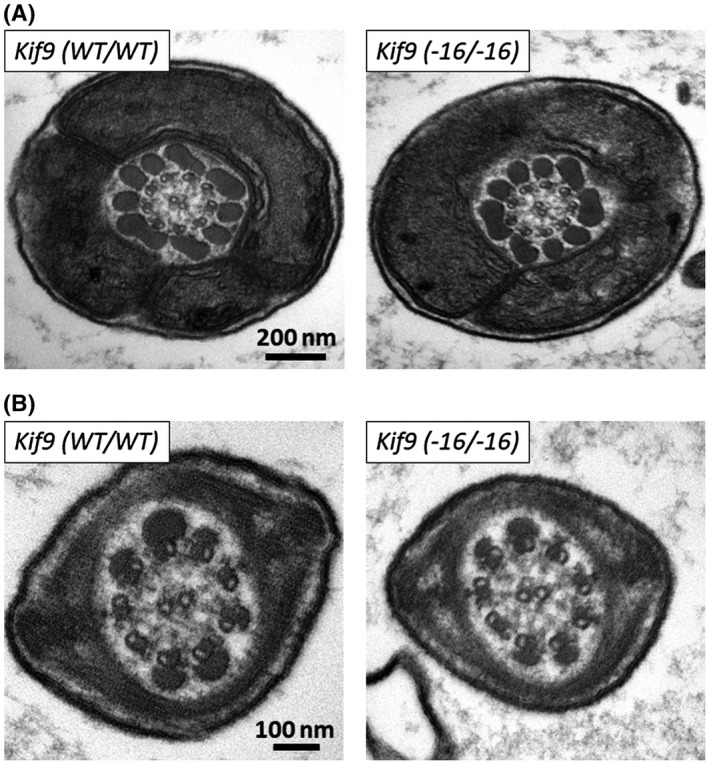
No obvious ultrastructural abnormalities were observed in the flagella of *Kif9^−16/−16^* mice. The midpiece (A) and principal piece (B) of spermatozoa within the cauda epididymis were observed with transmission electron microscopy

Because the deletion of axonemal proteins often leads to the disruption of axonemal structures,^12^, ^34^, ^35^, ^36^ we observed spermatozoa using transmission electron microscopy. No abnormalities were observed in both the midpiece (Figure 5A and Supplemental Figure S5) and principal piece (Figure 5B) of *Kif9^−16/−16^* mice, indicating that impaired sperm motility is not caused by obvious structural defects of the axoneme.

**Figure 6:**
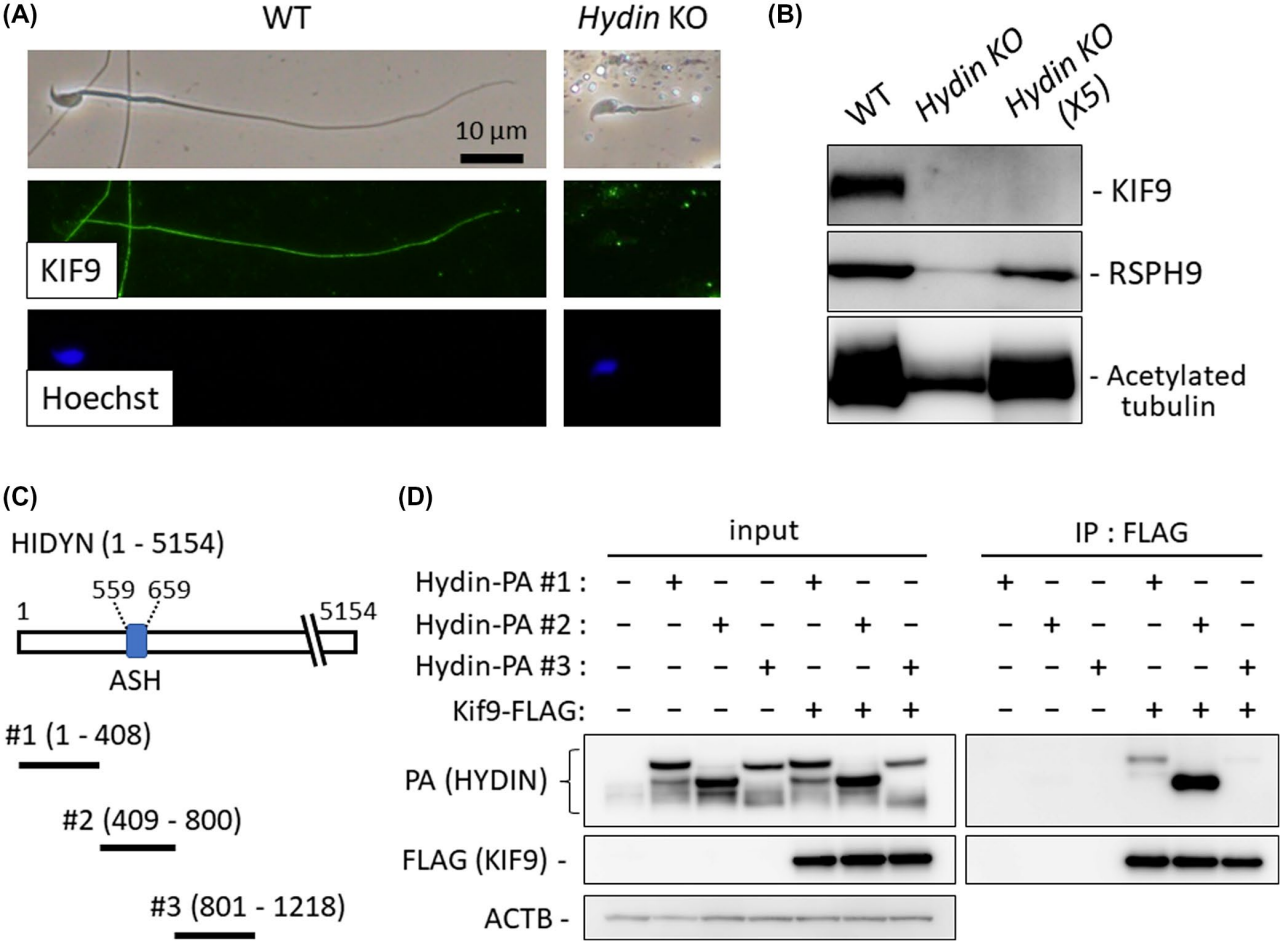
KIF9 disappeared in *Hydin* KO spermatozoa. A, *Hydin* KO spermatozoa obtained from the epididymis of *Hydin* KO chimeric mice were stained for KIF9. B, Western blotting analysis with the spermatozoa obtained from *Hydin* KO chimeric cauda epididymis. KIF9 was not detected even when five times the amount of protein was loaded (*Hydin* KO X5). In contrast, acetylated tubulin, indicating the presence of flagellum microtubules, and RSPH9, indicating the presence of radial spokes of the axoneme, were detected. C, Regions near the ASH domain of HYDIN were cloned for co‐immunoprecipitation analysis. D, *Hydin‐PA #1*, *Hydin‐PA #2*, or *Hydin‐PA #3* were co‐expressed with *Kif9‐FLAG* in HEK293T cells and immunoprecipitation with FLAG M2 antibody was performed. ACTB as control

In *Chlamydomonas*, KLP1 (*Chlamydomonas* ortholog of KIF9) is localized to the central pair of the axoneme and is associated with HYDIN, another central pair protein.^37^ In *Chlamydomonas*, *HYDIN* knockdown leads to a strong reduction in the amount of KLP1,^37^ suggesting an interaction between HYDIN and KLP1. In mice, HYDIN is localized to the central pair as well^28^; however, immunoprecipitation analysis of the KIF9‐HYDIN association could not be performed because it is difficult to solubilize KIF9 with mild lysis buffers (Figure 1D). Further, the lack of anti‐HYDIN antibodies makes it difficult to analyze HYDIN localization in the *Kif9* mutant mice. Therefore, we analyzed the localization of KIF9 in *Hydin* KO spermatozoa. *Hydin* KO causes hydrocephalus and lethality before sexual maturation, which hampers the analysis of the mature spermatozoa.^28^ Recently, we knocked out *Hydin* in fluorescently tagged ES cells using the CRISPR/Cas9 system.^26^ By making chimeric mice with these ES cells, we were able to analyze spermatozoa derived from *Hydin* KO ES cells and we found that HYDIN is essential for flagellum formation.^26^ To analyze if KIF9 is associated with HYDIN in mice, we analyzed KIF9 localization using these chimeric mice. Consistent with a previous study,^26^ *Hydin* KO spermatozoa exhibit short tails (Figure 6A). When we performed immunofluorescence with KIF9 antibody, no signals were detected in *Hydin* KO spermatozoa (Figure 6A). Further, when we performed Western blotting using the spermatozoa from the cauda epididymis in which the contribution of *Hydin* KO ES cells was high, no KIF9 bands were observed, although signals of acetylated tubulin and RSPH9, a protein localized in the radial spoke, were detected (Figure 6B). The disappearance of KIF9 in *Hydin* KO spermatozoa suggests that KIF9 may be associated with HYDIN and is localized to the central pair of the axoneme in mice.

To further analyze KIF9‐HYDIN interaction, we expressed FLAG‐tagged KIF9 and PA‐tagged HYDIN in HEK293T cells. Because HYDIN contains 5154 amino acids, which makes it difficult to clone the whole *Hydin* sequence, we focused on the ASPM‐SPD2‐Hydin (ASH) domain. The ASH domain is found in cilia‐ or centrosome‐associated proteins and is shown to interact with a different kinesin, KIF13B.^38^ Immunoprecipitation analysis revealed that KIF9 bound to the region containing the ASH domain (HYDIN #2) and weakly bound to the N‐terminus region of HYDIN (#1), but not to region #3 (Figure 6C,D). These results suggest that KIF9 could bind to the N‐terminus region of HYDIN that contains the ASH domain.

## 4 DISCUSSION

In this study, we revealed that KIF9 is localized to the mouse flagellum. Further, KIF9 was detected in the SDS soluble fraction, suggesting that KIF9 is associated with the axoneme. Because KIF9 disappeared in *Hydin* KO spermatozoa and interaction of KIF9 and HYDIN was confirmed with co‐immunoprecipitation assay, it is likely that KIF9 is localized to the central pair of the axoneme, consistent with *Chlamydomonas*.^5^, ^6^

By mutating *Kif9* in mice, we revealed that KIF9 is important for the progressive motility of spermatozoa and normal male fertility. *Kif9* mutant mice were not completely infertile likely because there are variations in the motility of individual spermatozoa and the spermatozoa with good motility could fertilize oocytes. Detailed analysis of flagellar motility of *Kif9* mutant mice showed that waveform patterns are asymmetric, indicating that switching of microtubule sliding is impaired. In *Chlamydomonas*, HYDIN is localized to the C2 microtubule of the central pair and is thought to be essential for the switch in bending direction by regulating dynein arm activity.^37^ By interacting with HYDIN through the ASH domain, KIF9 may also be involved in the switching. KIF9 possesses a motor domain in the C‐terminus and a previous study suggests that KIF9 possesses motor activity.^30^ It remains to be determined if the motor activity of KIF9 is involved in the directional switch in bending.

KIF9 belongs to the kinesin 9 family that contains another kinesin, KIF6.^4^ Northern blot analysis showed that *Kif6* is expressed in mouse testis^8^; however, *Kif6* mutant mice exhibited hydrocephalus leading to postnatal lethality,^39^ which makes it difficult to analyze KIF6 function in mature spermatozoa. The milder phenotype of *Kif9* mutant mice compared to *Hydin* KO mice, such as subfertility, no abnormalities in axonemal ultrastructures, or no overt hydrocephalus, may be due to the compensation by KIF6. It is also possible that KIF9 plays more specific roles in regulating sperm flagella, rather than regulating ciliary motility that exhibits different waveform patterns from flagella.

There are studies showing that kinesins play roles in spermiogenesis through intraflagellar transport or intramanchette transport.^40^, ^41^, ^42^ Although we cannot exclude the possibility that KIF9 is involved in these transports, which is important for regulating sperm motility, we could not observe any abnormalities in ultrastructures with transmission electron microscopy. Other KIFs may be involved in these transports during spermiogenesis. It is noteworthy to mention that we also mutated *Kif2b* that is expressed strongly in the testis; however, the mutant male mice exhibited normal fertility.^43^

In summary, we reveal that *Kif9*‐mutant male mice exhibit impaired sperm motility and male subfertility. Because *Kif9* is conserved in humans, revealing how KIF9 regulates flagellar motility may lead to better treatment for individuals with asthenozoospermia.

## CONFLICT OF INTEREST

The authors declare no conflicts of interest.

## AUTHOR CONTRIBUTIONS

H. Miyata and M. Ikawa designed the research; H. Miyata, K. Shimada, A. Morohoshi, S. Oura, T. Matsumura, Z. Xu, and Y. Oyama performed the research; H. Miyata, K. Shimada, A. Morohoshi, S. Oura, T. Matsumura, Z. Xu, Y. Oyama, and M. Ikawa analyzed the data; H. Miyata and M. Ikawa wrote the paper.
