# Supplementary material for: Testis‐enriched kinesin KIF9 is important for progressive motility in mouse spermatozoa
Source: FASEB J. 2020 Feb 19;34(4):5389–400. doi: 10.1096/fj.201902755R (PMC7136151; doi:10.1096/fj.201902755R)
Supplement: Supplementary file 1 [file FSB2-34-5389-s001.docx]

**Supplemental Figure S1**

**
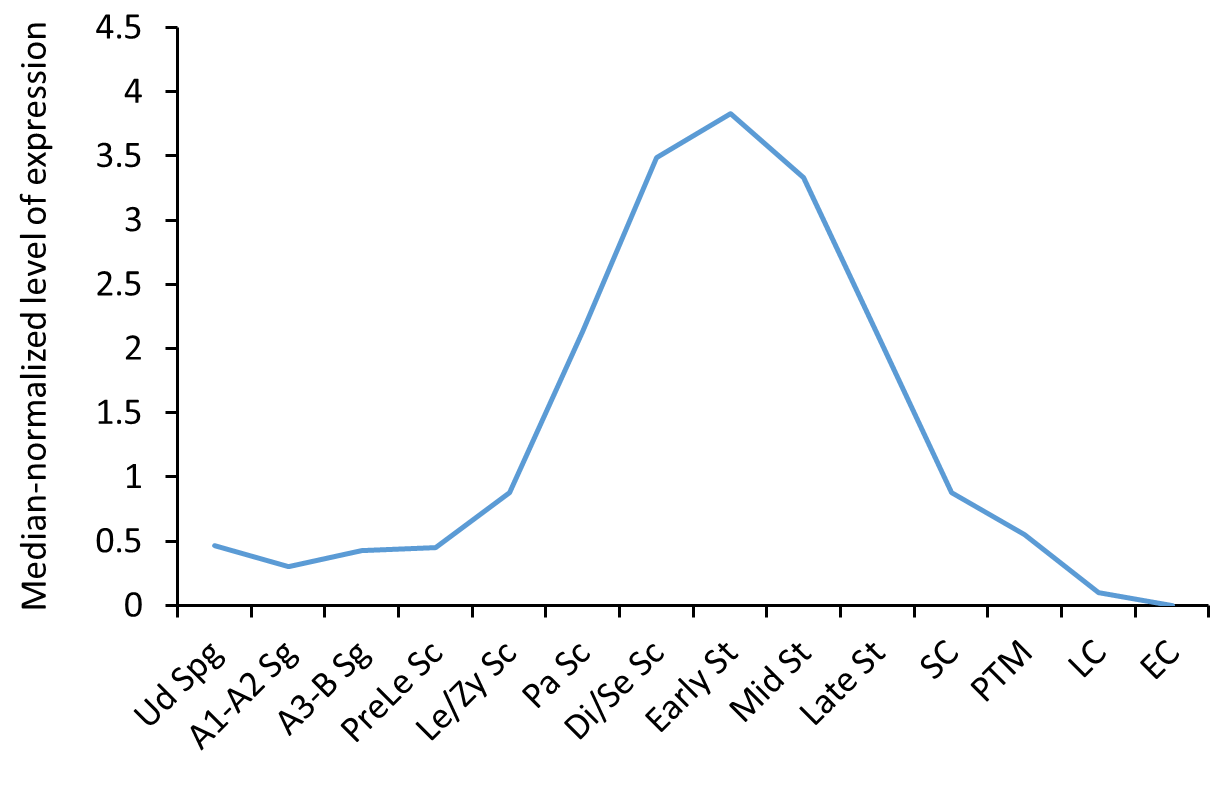
**

**Supplemental Figure S1. *In silico* data analysis of *Kif9* expression in mouse testis**

*Kif9* is expressed in spermatocytes and round spermatids. Ud Spg: undifferentiated spermatogonia, A1-A2 Sg: A1-A2 differentiating spermatogonia, A3-B Sg: A3-A4-In-B differentiating spermatogonia, PreLe Sc: Preleptotene spermatocytes, Le/Zy Sc: Leptotene/Zygotene spermatocytes, Pa Sc: Pachytene spermatocytes, Di/Se Sc: Diplotene/Secondary spermatocytes, Early St: Early round spermatids, Mid St: Mid round spermatids, Late St: Late round spermatids, SC: Sertoli cells, PTM: Peritubular myoid cells, LC: Leydig cells, EC: Endothelial cells.

**Supplemental Figure S2**

**
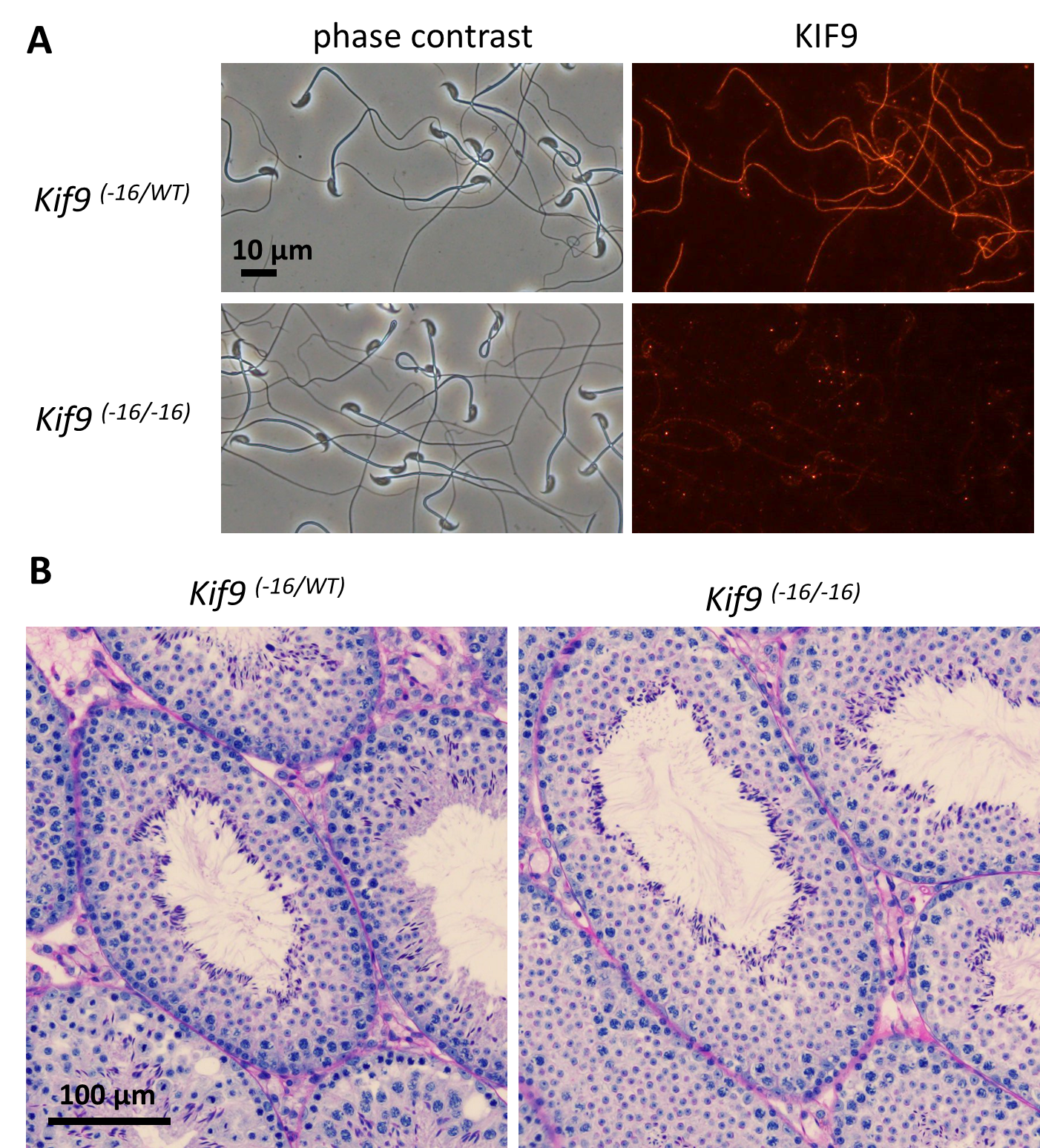
**

**Supplemental Figure S2. KIF9 localization in spermatozoa and testis sections**

(A) Immunofluorescence analysis for KIF9. KIF9 was not detected in *Kif9^-16/-16^* mice.

(B) PAS staining of testes. No abnormalities were found in *Kif9^-16/-16^* mice.

**Supplemental Figure S3**

**
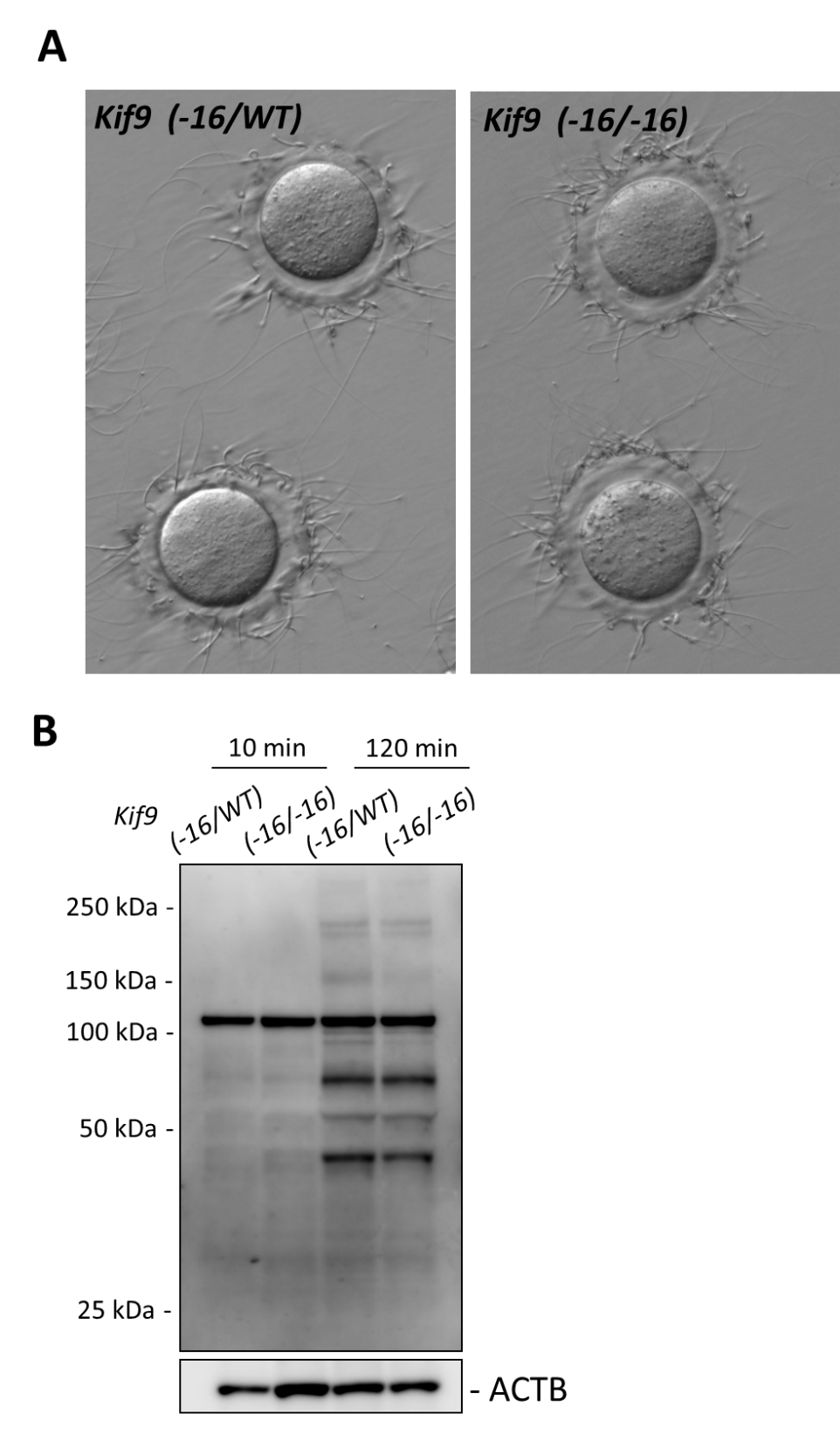
**

**Supplemental Figure S3. ZP binding assay and tyrosine phosphorylation**

(A) Spermatozoa from *Kif9^-16/-16^* mice are able to bind to the ZP.

(B) Phosphorylation status of tyrosine residues of sperm proteins. ACTB as control.

**Supplemental Figure S4**

**
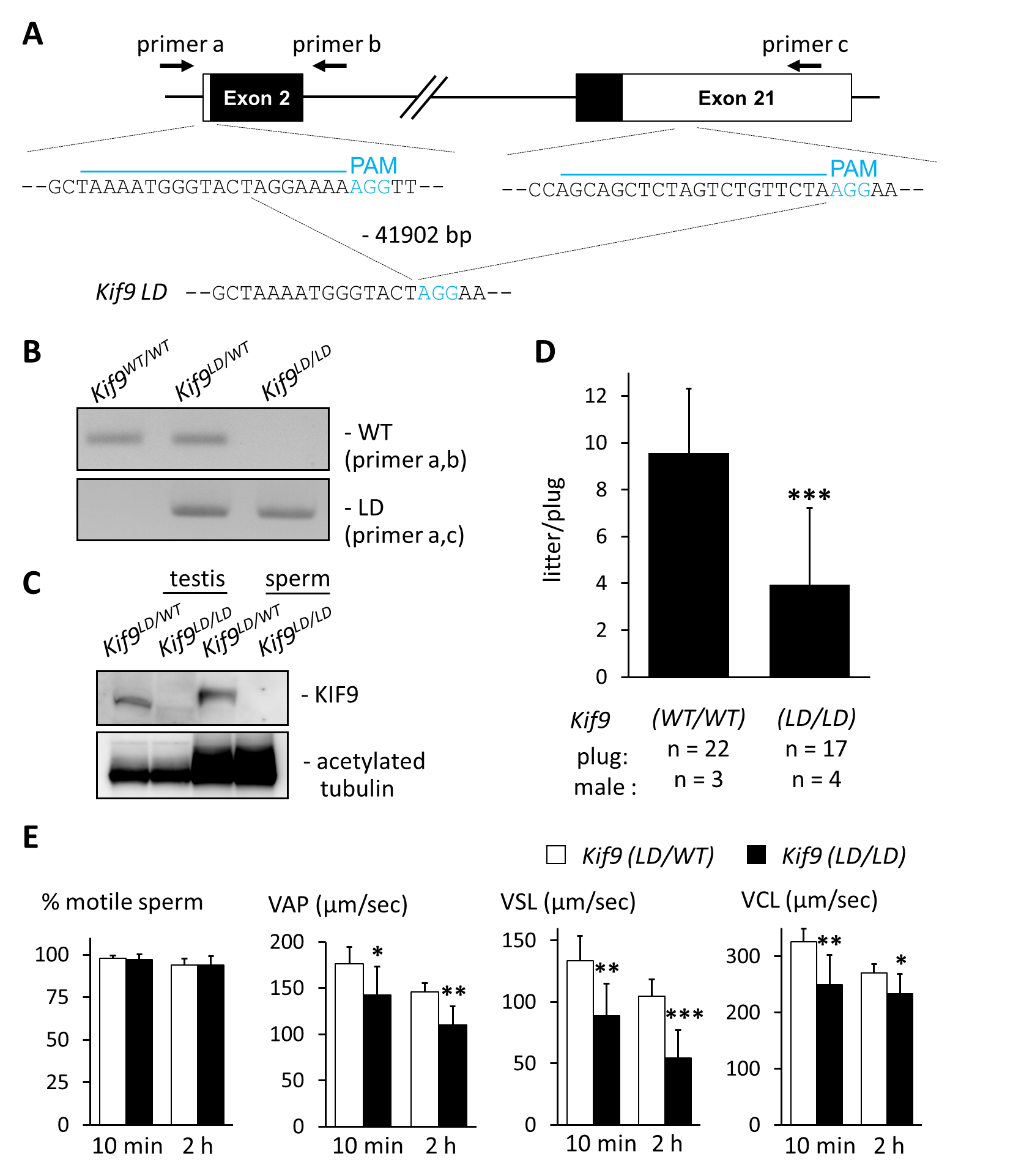
**

**Supplemental Figure S4. Generation and analysis of *Kif9* large deletion mice**

(A) CRISPR/Cas9 targeting scheme. gRNAs were designed within exon 2 and exon 21. Cyan characters indicate PAM (protospacer adjacent motif) sequence. 41902 bp nucleotides were deleted.

(B) Genotyping with PCR using primers described in (A). Primers ‘a’ and ‘b’ for the WT allele and primers ‘a’ and ‘c’ for the LD allele.

(C) Protein expression of KIF9 in testis and cauda epididymal spermatozoa. Acetylated tubulin as a loading control.

(D) Number of litters born per plug detected. n=3 males for *Kif9^WT/WT^* and n=4 males for *Kif9^LD/LD^* mice.

(E) Sperm motility was analyzed using a computer assisted sperm analysis system 10 minutes and 2 hours after incubation. n=6 males for *Kif9^LD/WT^* and n=7 males for *Kif9^LD/LD^* mice.

**Supplemental Figure S5**


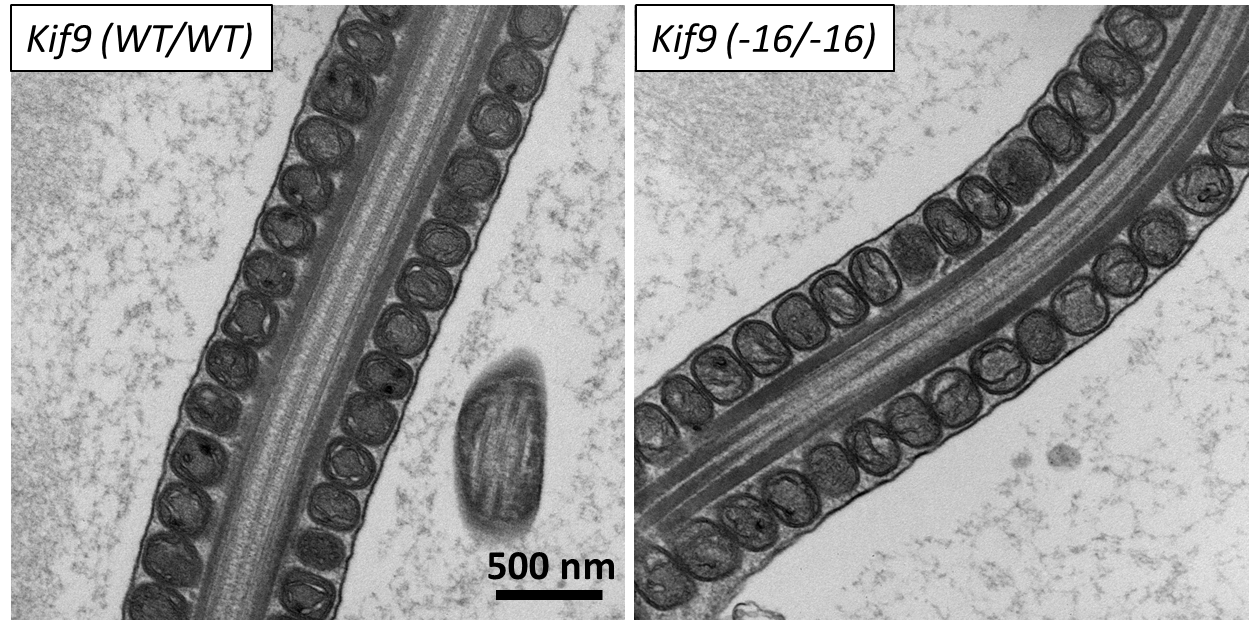


**Supplemental Figure S5. TEM analysis of spermatozoa**

Longitudinal sections of the midpieces. No structural abnormalities were observed in *Kif9^-16/-16^* mice.

**Supplemental movie S1. Sperm motility of *Kif9^-16/WT^* mice**

Sperm motility was videotaped at 50 frames per second 2 hours after incubation. The movie is played at 25 frames/second (1/2 speed).

**Supplemental movie S2. Sperm motility of *Kif9^-16/-16^* mice**

Sperm motility was videotaped at 50 frames per second 2 hours after incubation. The movie is played at 25 frames/second (1/2 speed).
